# Sheath Rupture in Figure‐Of‐Eight Suturing: A Case Report and Risk Reduction Strategies

**DOI:** 10.1002/ccr3.72934

**Published:** 2026-06-12

**Authors:** Takahiro Kobayashi, Yasushi Oginosawa, Yuki Nakamura, Taro Miyamoto, Yasunobu Yamagishi, Katsuhide Hayashi, Kazunobu Kawakami, Masaharu Kataoka

**Affiliations:** ^1^ Department of Cardiology University of Occupational and Environmental Health Kitakyushu Japan; ^2^ Department of Cardiology Kumamoto Rosai Hospital Yatsushiro Kumamoto Japan

**Keywords:** catheter ablation, figure‐of‐eight suture, hemostasis, preventive countermeasures, sheath injury

## Abstract

To prevent sheath perforation during figure‐of‐eight suturing, operators should optimize needle placement and suture tension, avoiding proximal sheath puncture. Polyether block amide sheath usage and intraluminal device retention during suturing provide additional protection. Standardized training and prompt suture removal upon resistance during sheath withdrawal may enhance safety.

## Graphical abstract

Risk reduction strategies for sheath rupture during figure‐of‐eight suturing include appropriate needle placement, controlled suture tension, avoidance of proximal sheath puncture, use of polyether block amide sheaths, and retention of an intraluminal device during suturing.

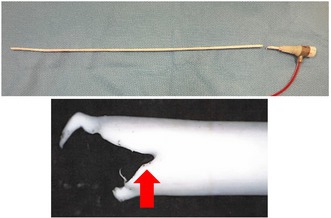

## 1 Introduction

**FIGURE 1:**
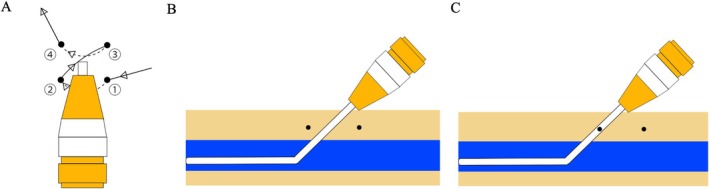
Schematic representation of the correct suturing and injury mechanism. (A) and (B) are schematic representations of the correct FoE suture, and (C) illustrates the mechanism of sheath injury. (A) is the top view, whereas (B) and (C) are transverse views. Initially, the needle is inserted from the inferior side of the sheath insertion site, traversing from the medial to the lateral aspect to suture the subcutaneous tissues. Subsequently, the superior aspect of the sheath insertion site is sutured in a similar manner, resulting in a “figure‐of‐eight” configuration. C illustrates an instance of an erroneous technique, wherein the suture needle penetrates the sheath at a right angle, leading to sheath damage. FoE, figure‐of‐eight.

Electrophysiological studies and catheter ablation are the cornerstones of arrhythmia treatment. Technological advances have improved the effectiveness of the procedure; however, the management of vascular access sites remains critical. Deshmukh et al. reported that vascular complications occur in 1.53% of catheter ablation procedures [1]. Conventional hemostatic procedures require prolonged manual compression, which can be uncomfortable for patients and require prolonged immobilization. To address these limitations, figure‐of‐eight (FoE) suturing and vascular closure devices have been introduced as alternatives to expedite hemostasis and promote early ambulation [2, 3, 4]. The FoE suture technique is preferred as it is simple and cost‐effective. Despite its advantages, FoE suturing is associated with risks such as sheath rupture, especially due to improper needle placement, excessive suture tension, or material failure (Figure 1). To the best of our knowledge, this is the first report of direct complications associated with FoE suturing. In this case report, we focus on the complications of sheath rupture, explore its underlying mechanisms, and propose risk‐reducing measures.

## 2 Case History/Examination

### 2.1 Patient Background and Procedure

A 44‐year‐old male patient presented with an incidental Brugada type I electrocardiographic pattern. The patient had no family history of ventricular fibrillation or syncope. However, he underwent electrophysiological testing to determine the necessity for an implantable cardioverter‐defibrillator [5]. The patient had no significant medical history, and the pre‐procedure evaluation yielded no notable findings. The procedure was performed under local anesthesia with ultrasound‐guided femoral vein access, and two 5 Fr, 25 cm sheaths were inserted into the femoral veins. Electrophysiological studies were completed without any complications.

### 2.2 Complication Presentation

**FIGURE 2:**
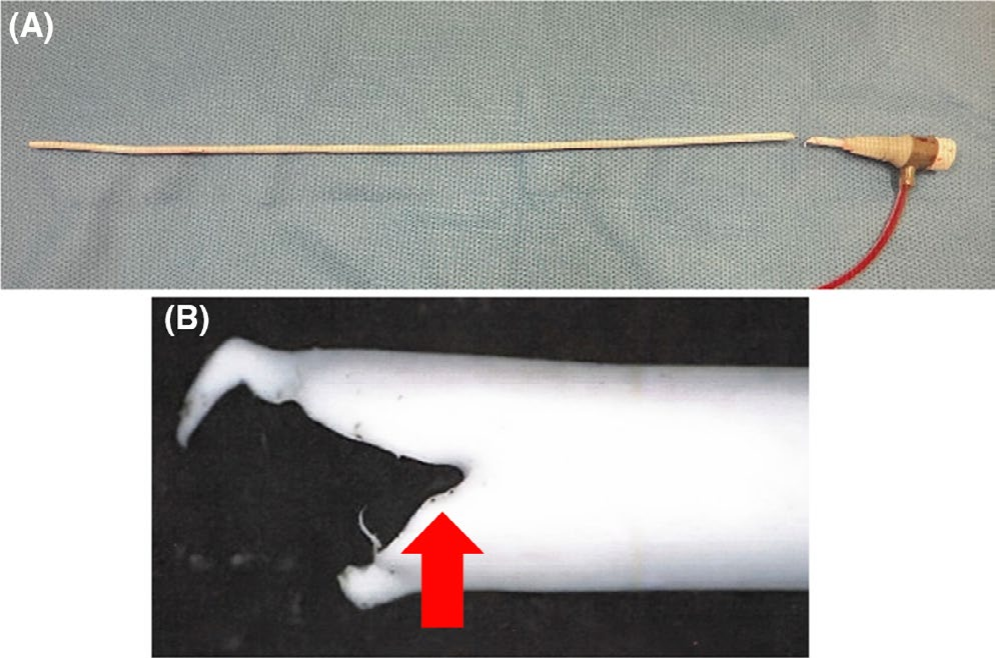
Sheath rupture process and ruptured sheath cross section. (A) The sheath is ruptured 10 mm from the root. (B) The magnified cross section of the sheath is shown. The arrow shows the deformation of the sheath that is believed to have occurred after the suture needle penetrated the sheath. The upper part of the sheath above the deformed area is assumed to be damaged and torn by the suture. A reproduction of the experiment is shown in Figure 3.

The following materials were used for hemostasis after the procedure: a 1/2 circle, 33 mm diameter suture needle and 1–0 silk FoE suture thread. Initially, there were no complications during suturing; however, mild resistance was observed during sheath removal, followed by an abrupt rupture of the sheath wall and subsequent partial detachment (Figure 2).

## 3 Differential Diagnosis, Investigations, and Treatment

### 3.1 Differential Diagnosis

The sudden sheath rupture during removal required immediate assessment of potential causes:

– Mechanical failure due to material defect.
– Excessive suture tension causing structural compromise.
– Inadvertent needle puncture creating initial perforation.
– Shearing forces during removal.

### 3.2 Investigation and Treatment

The use of a thicker sheath and snare was considered a retrieval method for the fractured sheath. However, given the length of the fractured sheath and its proximity to the femoral puncture site, a more distal venous approach was considered necessary. Consequently, emergency surgical intervention was performed, involving an incision of the groin, exposure of the femoral vein under direct vision, and removal of the sheath fragment using a snare.

## 4 Conclusion and Results (Outcome and Follow‐Up)

### 4.1 Immediate Outcome

The emergency surgical procedure was successfully completed, and hemostasis was achieved without further complications. The sheath fragment was completely removed from the femoral vein under direct visualization.

### 4.2 Follow‐Up

The patient was discharged on postoperative Day 3 without any residual complications. Post‐operative recovery was uneventful, and no long‐term sequelae were observed.

### 4.3 Clinical Conclusion

FoE suturing is an effective method for vascular closure, but presents risks, including sheath rupture. This case emphasizes the importance of correct needle placement, controlled suture tension, and appropriate selection of sheath material. Retaining the intraluminal device during suturing provides an additional protective measure. Standardized protocols and enhanced operator training are essential to mitigate complications. Further research is necessary to optimize hemostasis strategies for catheter ablation.

## 5 Discussion

**FIGURE 3:**
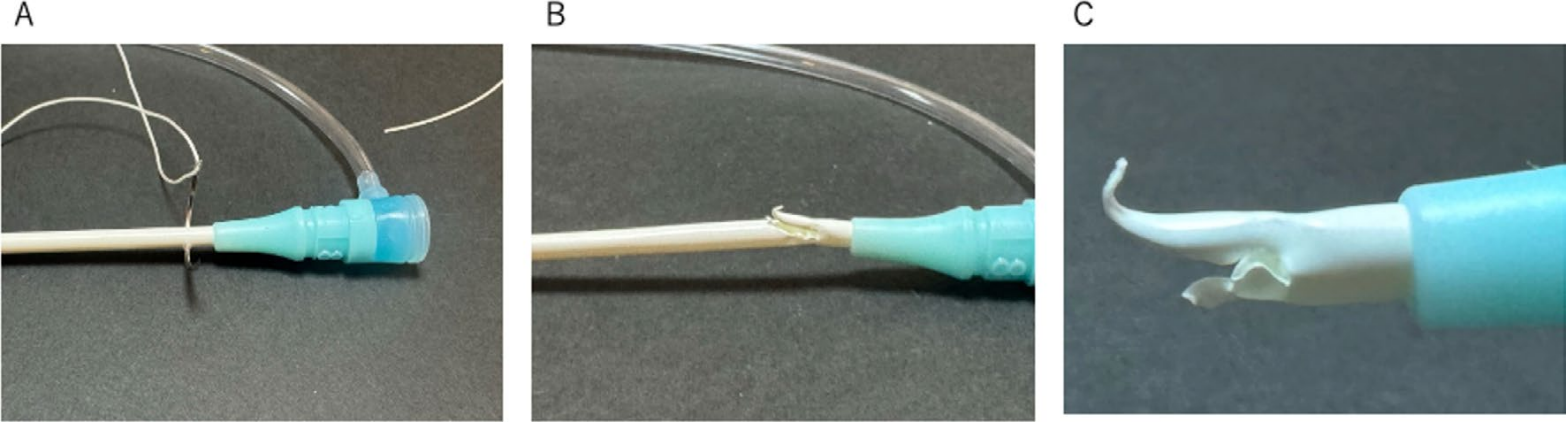
Reproduction of the mechanism of sheath damage during FoE suturing. (A) shows how the suture needle accidentally penetrates the sheath, and the subsequent tension from the suture thread causes the penetration site to expand (B), eventually leading to sheath rupture (C). This figure visually illustrates the procedural precautions that can be taken to reduce the risk of sheath injury.

This case highlights the risk of sheath rupture as a significant complication of FoE suturing. The analysis suggested that inadvertent needle penetration created a perforation that expanded under suture tension, leading to a full rupture. The experimental data confirm that minor sheath defects propagate under mechanical stress (Figure 3).

Needle insertion position is a critical risk factor for perforation. The perforation risk increases when the needle is placed too close to the puncture site, because the needle trajectory may pass through the sheath wall, rather than the surrounding subcutaneous tissue. Therefore, it is imperative to maintain a safe distance from the sheath to mitigate the risk of injury. This distance should be equivalent to half the suture needle length for sheath insertion at 45°, or half the radius for insertion at 60°. The sheath material characteristics are also significant. In our experiments, the polyether block amide sheaths used during ablation exhibited superior elasticity and resilience compared with polymer sheaths, thereby preventing the needle from penetrating the sheath wall. Catheter or dilator retention during suturing may serve as a mechanical buffer by supporting the sheath lumen and reducing deformation during needle passage.

A conceptually similar phenomenon has been described with the Perclose ProGlide suture‐mediated closure system, in which the closure mechanism inadvertently entrapped an adjacent intraluminal device during deployment, occasionally requiring surgical exposure for release [6]. Although the device and procedural setting differ from those of FoE suturing, the underlying mechanism is similar in that a closure‐related needle or suture may unintentionally capture an adjacent intraluminal structure when multiple devices are present in a confined access field. The recognition of abnormal resistance during sheath removal is therefore critical, as this may represent early evidence of suture‐related sheath capture and should prompt immediate reassessment, rather than forceful withdrawal. Accordingly, several practical preventive strategies may help to reduce this risk. Operators should control the needle insertion position and avoid suturing proximal to the insertion site. In addition, intraluminal devices should be held during suturing whenever possible. Standardized procedural protocols and operator training may further reduce avoidable technical errors.

## Author Contributions

**Takahiro Kobayashi:** conceptualization, data curation, formal analysis, investigation, methodology, visualization, writing – original draft, writing – review and editing. **Yasushi Oginosawa:** conceptualization, formal analysis, investigation, methodology, supervision, writing – review and editing. **Yuki Nakamura:** formal analysis, writing – review and editing. **Taro Miyamoto:** formal analysis, writing – review and editing. **Yasunobu Yamagishi:** formal analysis, writing – review and editing. **Katsuhide Hayashi:** formal analysis, writing – review and editing. **Kazunobu Kawakami:** formal analysis, investigation, methodology, writing – review and editing. **Masaharu Kataoka:** project administration, supervision, writing – review and editing.

## Funding

The authors have nothing to report.

## Ethics Statement

The authors have nothing to report.

## Consent

Written informed consent was obtained from the patient for the preparation and publication of this case report.

## Conflicts of Interest

The authors declare no conflicts of interest.

## Data Availability

All data underlying the results are available as part of the article, and no additional source data are required.
